# Visualization analysis of research frontiers and trends in the treatment of sciatic nerve injury

**DOI:** 10.3389/fneur.2024.1378689

**Published:** 2024-05-22

**Authors:** Yan Wang, Yahui Wang, Lijie Lv, Tianyi Li, Yan Wang, Fei Pei

**Affiliations:** ^1^Department of Rehabilitation Medicine and Physical Therapy, Graduate School, Heilongjiang University of Chinese Medicine, Harbin, China; ^2^Rehabilitation Center, The Second Affiliated Hospital of Heilongjiang University of Traditional Chinese Medicine, Heilongjiang University of Traditional Chinese Medicine, Harbin, China

**Keywords:** sciatic nerve injury, nerve regeneration, nerve repair, neuroprotection, therapy, treatment, visual analysis, web of science

## Abstract

**Objective:**

To visualize and analyze the literature related to sciatic nerve injury treatment from January 2019 to December 2023, and summarize the current status, hotspots, and development trends of research in this field.

**Methods:**

Using CiteSpace and VOSviewer software, we searched the Web of Science database for literature related to the treatment of sciatic nerve injury. Then we analyzed and plotted visualization maps to show the number of publications, countries, institutions, authors, keywords, references, and journals.

**Results:**

A total of 2,653 articles were included in the English database. The annual number of publications exceeded 230, and the citation frequency increased yearly. The United States and China were identified as high-influence nations in this field. Nantong University was the leading institution in terms of close cooperation among institutions. The authors Wang Yu had the highest number of publications and were highly influential in this field. Keyword analysis and reference Burst revealed a research focus on nerve regeneration and neuropathic pain, which involve regenerative medicine and neural tissue engineering. Chronic pain resulting from sciatic nerve injury often manifests alongside anxiety, depression, cognitive-behavioral disorders, and other issues. Interventions such as stem cells, electrical stimulation, electroacupuncture, total joint replacement, pharmacological interventions, gene therapy, nerve conduits, chitosan scaffolds, and exercise promote nerve repair and alleviate pain. Schwann cells have been the focus of much attention in nerve repair and regeneration. Improving the outcome of sciatic nerve injury is a current research challenge and focus in this field. Based on keyword Burst, nerve conduits and grafts may become a potential research hotspot in the treatment of sciatic nerve injury.

**Conclusion:**

This visual analysis summarizes research trends and developments of sciatic nerve injury treatment and predicts potential research frontiers and hot directions.

## Introduction

The sciatic nerve is the longest and thickest nerve in the human body. It originates from the lumbosacral plexus and divides into two terminal branches: the tibial nerve and the common peroneal nerve, which innervate the motor and sensory functions of the lower limbs. Sciatic Nerve Injury (SNI), a common peripheral nerve injury, is often associated with hip dislocation, acetabular fracture, and other traumatic injuries (1, 2). Despite the availability of various supportive therapies, surgical and non-surgical interventions, the clinical manifestations of neurological dysfunction with intermittent chronic pain persist due to the slow recovery of sciatic nerve injury and the histological properties of neurons. However, the recovery of target organ function after sciatic nerve injury remains challenging. Inappropriate treatment not only fails to guarantee functional recovery, but may also result in irreversible neuronal atrophy, leading to limb paralysis and severe disability in patients (3, 4). Chronic neuropathic pain resulting from peripheral nerve injury is a challenging clinical issue. Existing treatments only provide partial relief of symptoms and are costly, placing a significant burden on patients and society. Additionally, patients often experience accompanying symptoms such as depression, anxiety, and insomnia, which can have a detrimental effect on their physical and mental health (5–7). The repair, regeneration, and pain relief associated with peripheral nerve injury remain essential topics for current research.

To date, only one study has been identified that utilizes CiteSpace software to visualize the repair and protection of sciatic nerve injuries, and it dates back a decade. Therefore, this paper utilizes CiteSpace 6.2R6 software and VOSviewer 1.6.20 to visualize and analyze the relevant literature in this field over the past 5 years. The aim is to summarize the development status, research hotspots, and shortcomings, providing a reference for current and future studies in sciatic nerve injury treatment.

## Data and methods

### Data source

Web of Science database was retrieved Web of Science core Collection, and the search period was set from January 2019 to December 2023 using the following search strategy. TS = (“Sciatic nerve injury” OR“Sciatic nerve”) AND (“therapeutics” OR “therapy” OR “therapies” OR “treatment” OR “treatments” OR “repair” OR “regeneration” OR “protect”).Document.

Types = “Article OR Review Article”; Time Span = “2019–2023”; Languages = “English,” Index = “SCI-EXPANDED.”The search for data was completed on December 23, 2023, and 2,658 documents were retrieved and exported.

### Research methods

The English literature was downloaded and saved in the format of “full record with cited references” exported in “plain text,” and named as “download_x-x.txt” CiteSpace 6.2.R6 was used to remove the weights, resulting in the inclusion of 2,653 documents. For the analysis, CiteSpace was set to the time period of 2019–2023 with a default time partition of 1. The data was converted into a recognizable format and visualized in terms of the number of publications, countries, institutions, authors, cited journals, and keywords. Additionally, VOSviewer 1.6.20 software was used to highlight the attention of relevant core institutions, and authors in the field. The analysis type includes co-authorship and co-occurrence, the analysis unit of analasis involves institutions, authors, and keywords. The calculation method selection is full counting. Each dot in the graph represents a node type, with larger dots indicating a higher frequency or number. The connecting lines represent the citation relationship between each dot, with more connecting lines indicating closer cooperation relationships. To obtain a visual mapping, run the software and then manually adjust the threshold, size, color of nodes and labels, and the clarity of the color of connecting lines to enhance the clarity and esthetics of the image.

## Results

### Publication outputs and citation trends

**Figure 1 fig1:**
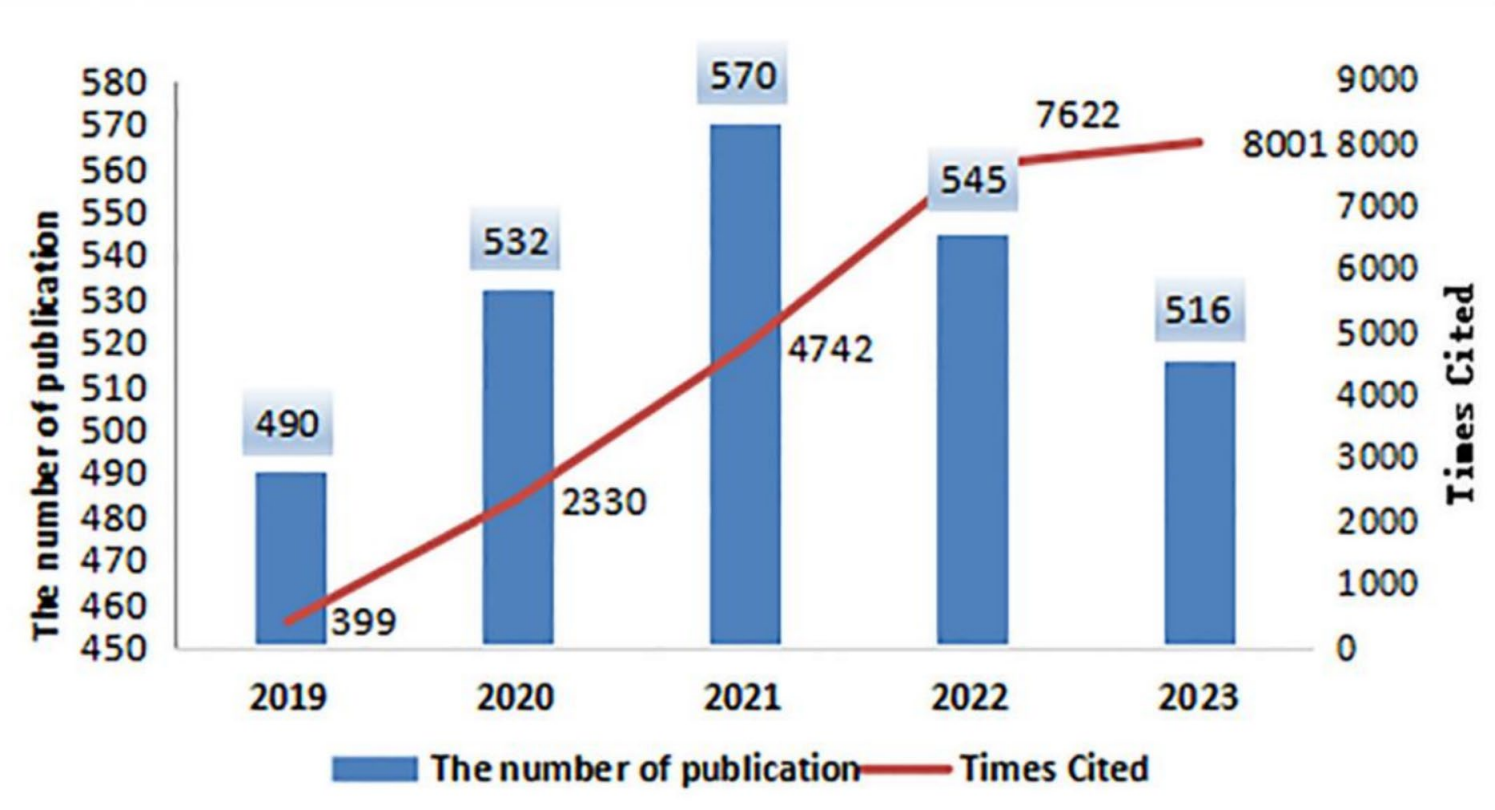
The number of annual publications and times cited.

The literature included in this study was statistically analyzed in terms of the number of articles published and the frequency of citations based on the time of publication, as shown in Figure 1. A total of 2,653 articles were published in the past 5 years, with an upward trend in the number of publications from 2019 to 2021, reaching a peak in 2021. The overall annual number of publications remained above 490 from 2021 to 2023, although there was a slight decrease in the number of publications. The total citation frequency was 23,224, increasing annually, with the highest frequency of 8,001 in 2023. The average number of citations per item was 8.74, and the H-index was 49.

### Countries and regions

**Figure 2 fig2:**
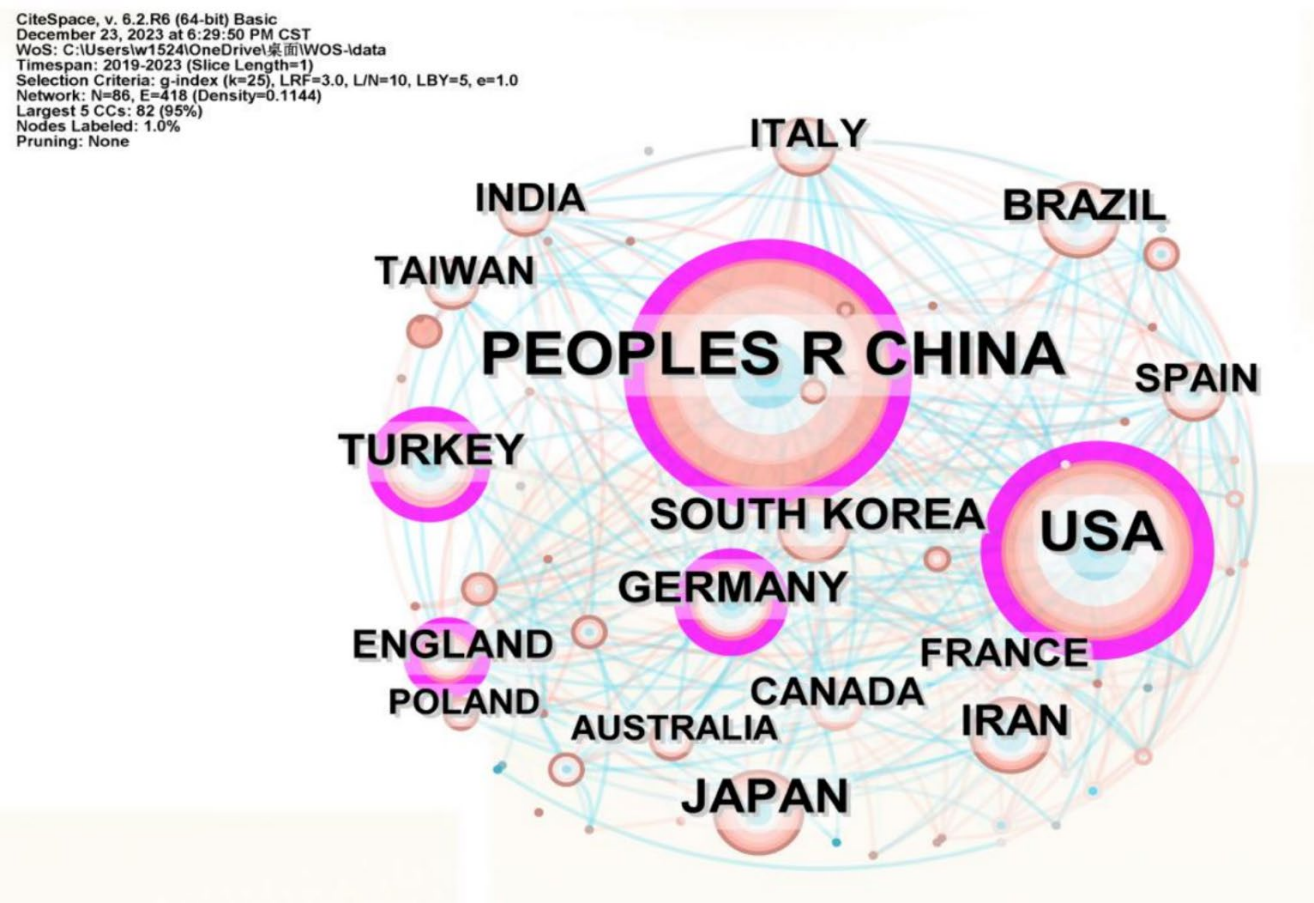
The figure shows the Country/regional cooperative network with relevant publications. The circles in the graph represent different countries, and the size of each circle is proportional to the number of articles published by that country. The size of the purple circle indicates the centrality of a country in the network, while the thickness of the connecting lines reflects the strength of collaboration between countries.

A total of 2,653 documents were included in the English database from 2019 to 2023. Utilizing CiteSpace 6.2.R6 software to analyze literature from the Web of Science database, as shown in Figure 2, the data on countries and regions collaboration in the field reveals a network with 75 nodes, 207 connections, and a network density of 0.0746. Currently, 75 countries have published articles related to the treatment of sciatic nerve injuries, with relatively weak interconnections among them. Refer to Table 1, which shows that 8 countries have published more than 100 articles, with China having the largest number of publications in this field, accounting for 35.39% of the total number of publications and having a centrality score of 0.24. The United States follows in publication volume but has the highest centrality score of 0.3. China and the United States also rank in the top two in total citations, underscoring their significant contributions and influence in the field of sciatic nerve injury repair and applied research.

**Table 1 tab1:** The top 8 most productive countries and regions with publications.

Rank	Country/regions	Counts	Proportion (%)	Centrality	Citations
1	China	939	35.39	0.24	9,402
2	The United States	552	20.81	0.30	5,604
3	Japan	159	5.99	0.02	968
4	Brazil	139	5.23	0.07	944
5	SouthKorea	121	4.56	0.04	918
6	Turkey	118	4.45	0.16	573
7	Iran	108	4.07	0.07	1,526
8	Germany	108	4.07	0.18	1,063

### Institutions

A total of 288 institutions have published articles on the treatment of sciatic nerve injury, with universities being the primary contributors. As shown in Table 2, which lists the top 10.

**Table 2 tab2:** Ranking of organizations by number of publications and centrality

Institution	Country	Publications	Centrality
Nantong university	China	125	0.13
Shanghai Jiao Tong university	China	72	0.18
Sun Yat Sen university	China	48	0.01
Peking university	China	47	0.11
Universidade de Sao Paulo	Brizlin	44	0.16
University of California system	United States	41	0.36
Harvard university	United States	40	0.30
Sichuan university	China	39	0.08
Nanjing medical university	China	36	0.10
Jilin university	China	35	0.08

institutions by publication volume, 7 of these are based in China. The top three institutions are Nantong University (125), Shanghai Jiao Tong University (72), and Sun Yat Sen University (8). Among them, two institutions are from the United States, the namely University of California System (9) and Harvard University (10). Although the number of publications of these two institutions is slightly less than that of some Chinese institutions, the centrality of these two institutions ranks the top two, respectively 0.36 and 0.30. All are high-impact institutions that are leading the way in this field of research. (refer to Figure 3). The co-occurrence time overlay Visualization of institutional publication volume is plotted using VOSviewer 1.6.20 (refer to Figure 3). The results indicate that domestic research institutions are more closely cooperating, with Nantong University having the most cooperation with other institutions. However, cooperation with foreign institutions is less frequent. Since April 2021, the Nanjing University of Chinese Medicine, Beijing University of Chinese Medicine, Southern Medical University, Shanghai University of Traditional Chinese Medicine, Dalian University of Traditional Chinese Medicine, and Guangzhou University of Traditional Chinese Medicine have demonstrated a growing interest in researching the treatment of sciatic nerve injuries. This indicates that major Chinese medicine institutes are focusing more on this field.

**Figure 3 fig3:**
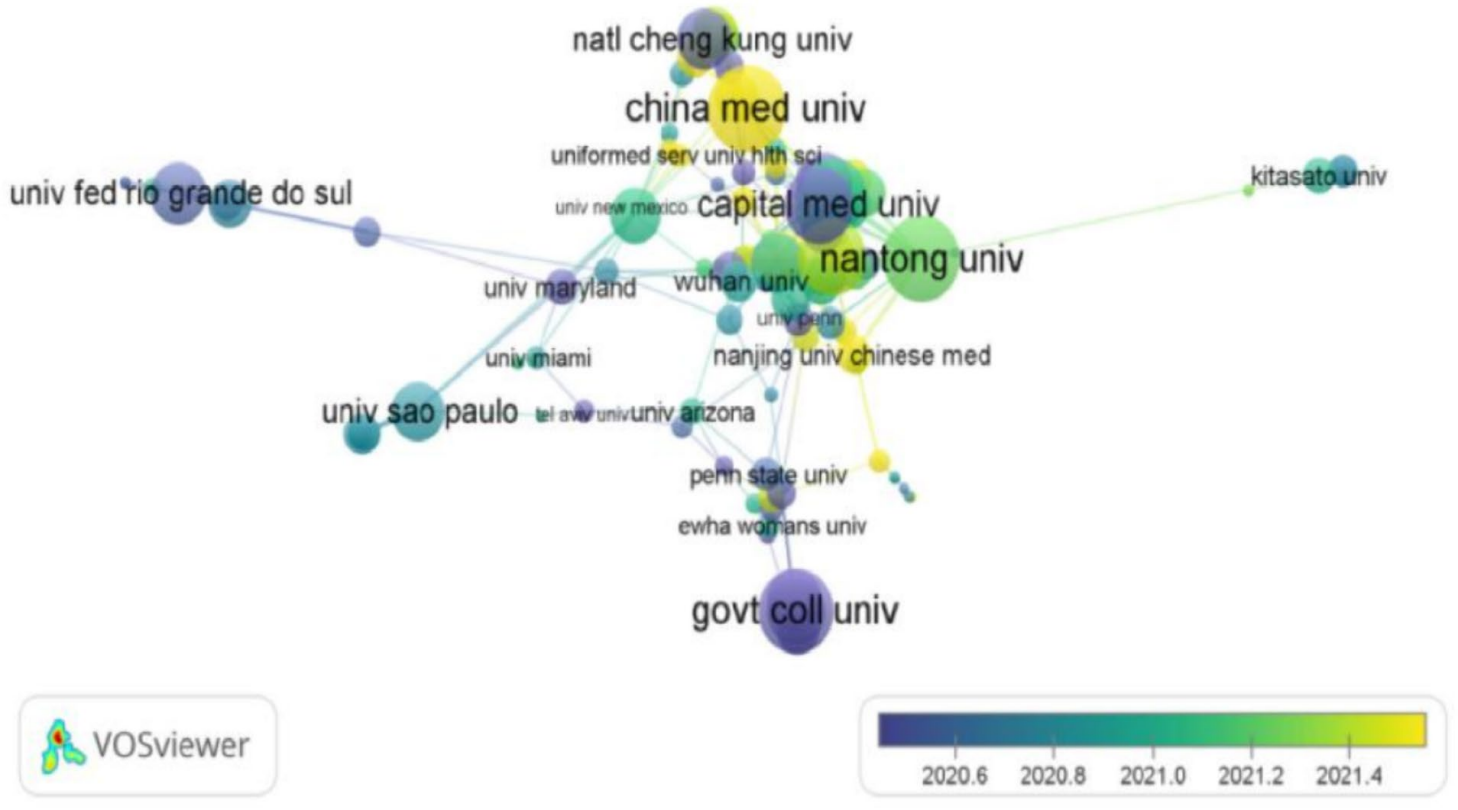
Institutional with co-occurrence relations shown as an overlay graph plotted. The circles represent keywords, and the size of the circles represents the frequency of keywords. The size of the circles is proportional to the frequency of keywords. Circles with the same color form a cluster group, and the connecting lines represent the connection between keywords.

### Co-authorship and co-citation author

**Figure 4 fig4:**
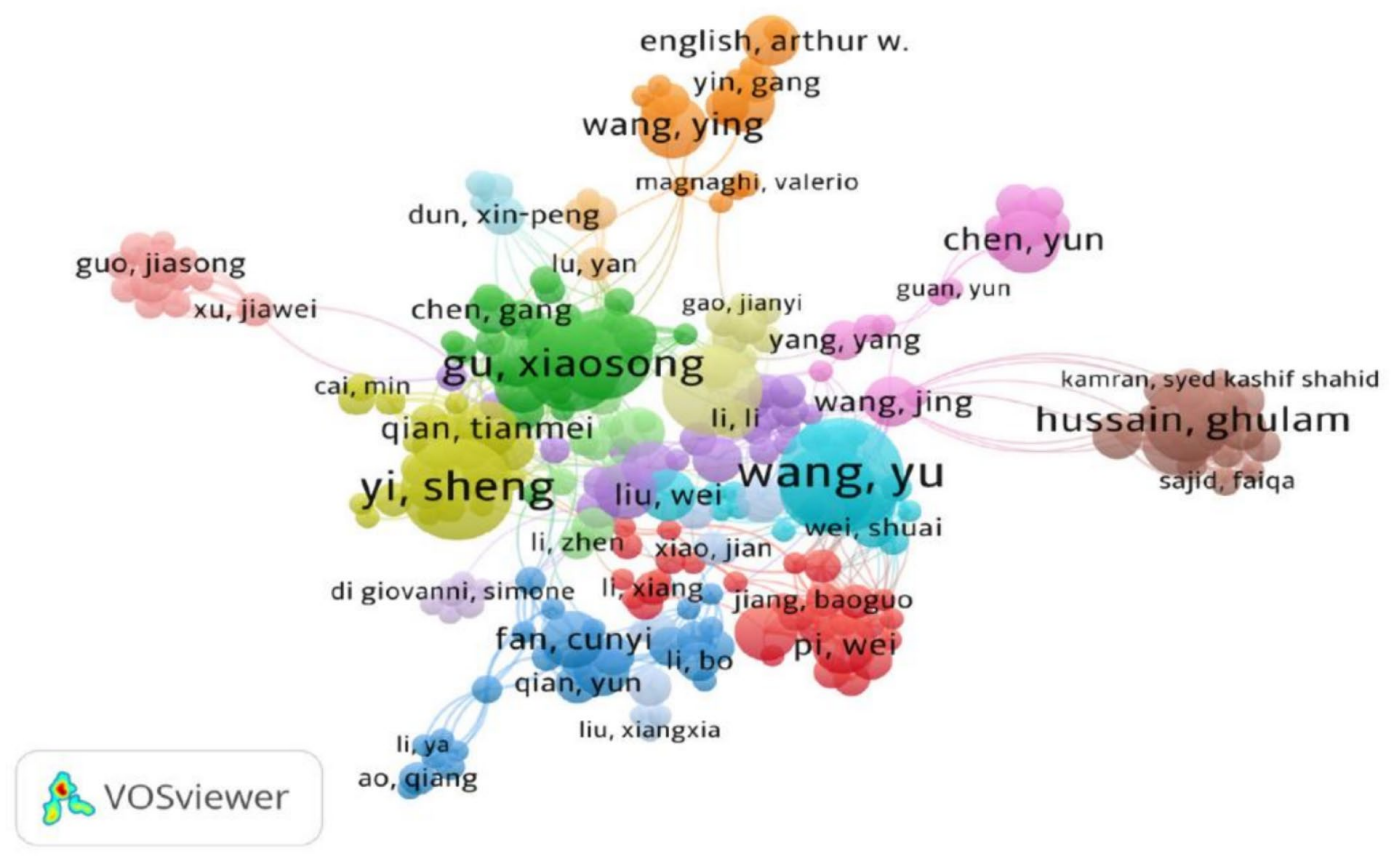
The shows the network diagram of Author Collaboration. In the map, each circle represents an author, which is proportional to the amount of papers published by the author. Circles of the same color represent the cooperative group formed between the authors, and the line represents the connection between each author, the thicker the line, the closer the co.

A total of 7,215 authors participated in 2653 documents. Table 3 shows that there were 7 authors with ≥10 publications, with Wang, Yu having the highest number of publications (11) and citation frequency (296). Price’s law was used to calculate the number of publications of core authors based on M = 0.749 × (Nmax)^1/2^ = 0.749 × 20 ^1/2^ ≈ 3.35, which indicates that authors with 4 publications are the core authors in the field of sciatic nerve injury research, and there are a total of 483 core authors. A collaborative co-occurrence network mapping of authors was produced using VOSviewer 1.6.20 (Figure 4), which only displays authors of publications with a publication volume of ≥4. The research team consists of 16 core authors, including Wang, Yu, Yi, Sheng, Hussain, and Ghulam. Typically, the team is composed of researchers from the same university, affiliated hospitals, or region.

**Table 3 tab3:** The top 7 most productive author from 2019 to 2023.

Author	Publications	Citations
Wang, Yu	20	296
Yi, Sheng	18	190
Gu, Xiaosong	16	152
Hussain, Ghulam	13	156
Mika, Joanna	12	196
Navarro,Xavier	12	275
Peng, Jiang	10	196

**Figure 5 fig5:**
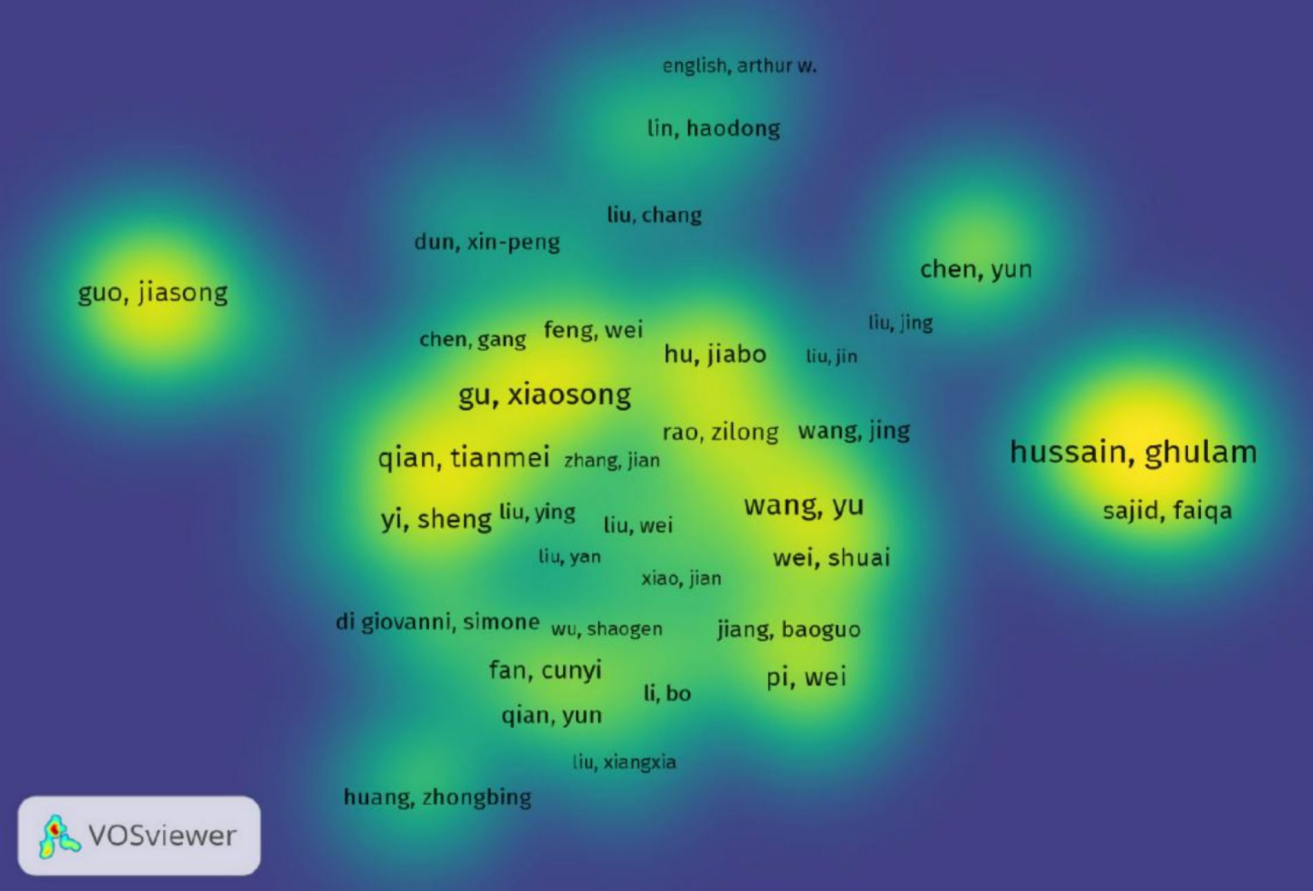
The shows a density mapping of author collaboration networks. In the atlas, areas with higher density of authors are closer to bright yellow, while those with lower density lean toward blue. The density is determined by the strength and significance of author connections in the surrounding area.

When using VOSviewer to create a density view of authors’ co-authorship, it is recommended to set the weights to total link strength, as shown in Figure 5. This view can effectively display the collaboration and research density among high-impact authors in important fields. In conjunction with Table 4, this view highlights the top 7 authors, each having more than 60 links with others. It is evident that Hussain, Ghulam possesses the highest number of links (80) and the highest H-index (12), followed by Rasul, Azhar (75) and the H-index (11).

**Table 4 tab4:** The top7 Total link strength author from 2019 to 2023.

Author	Total link strength	H-index
Hussain, Ghulam	80	28
Rasul, Azhar	75	20
Anwar, Haseeb	72	15
Ikeguchi, Ryosuke	72	7
Matsuda, Shuichi	68	17
Aoyama, Tomoki	67	13
Takamatsu, Kiyohito	61	6

### Keywords

#### Keywords co-occurrence

In bibliometrics, the frequency of keyword occurrences can reveal the hotspots and trends in a research field. In this study, 8 keywords directly related to the topic were excluded: sciatic nerve, injury, regeneration, repair, peripheral nerve injury, peripheral nerve, nerve regeneration, and peripheral nerve regeneration. The top 10 keywords are listed in Table 5, each appearing more than 160 times. Among them, neuropathic pain, expression, and Schwann cells are the most frequent, occurring 414, 396, and 357 times, respectively. With the highest centrality of 0.6, “expression” is considered a turning point or a key focus in the field.

**Table 5 tab5:** Keyword frequency and centrality.

Rank	Count	Keywords	Centrality	Keywords
1	414	Neuropathic pain	0.06	Expression
2	396	Expression	0.05	Axonal regeneration
3	357	Schwann cells	0.05	Mesenchymal stem cells
4	224	Activation	0.05	Neuropathic pain
5	214	Functional recovery	0.04	Animal models
6	188	Model	0.04	Chronic pain
7	172	Spinal cord	0.04	Hyperalgesia
8	171	Oxidative stress	0.04	Involvement
9	166	Mechanisms	0.04	Microglia
10	163	Rat	0.04	Neurotrophic factor

**Figure 6 fig6:**
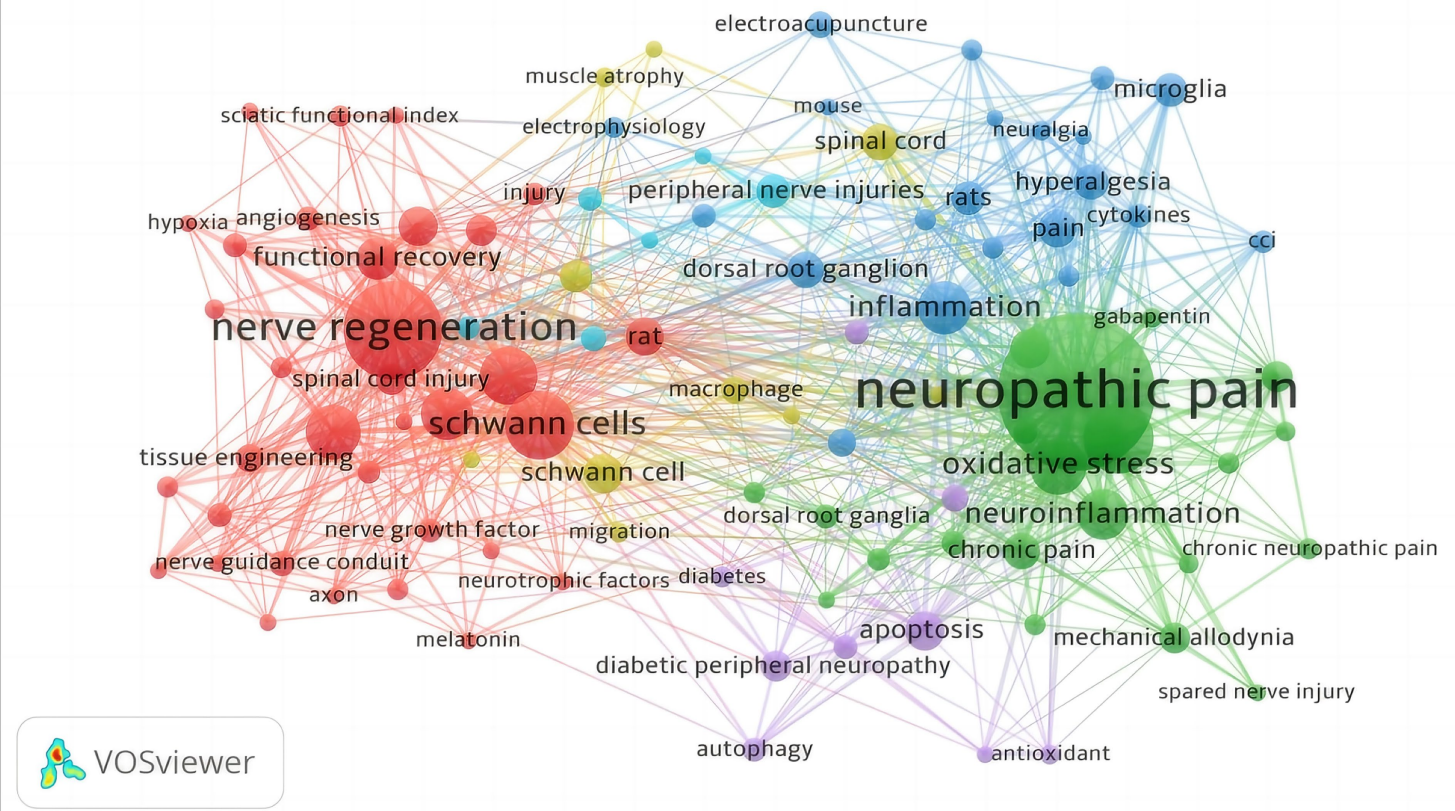
The shows the network diagram of Co-occurrence analysis of keywords. The circles represent keywords, and the size of the circles represents the frequency of keywords. The size of the circles is proportional to the frequency of keywords. Circles with the same color form a cluster group, and the connecting lines represent the connection between keywords.

By using VOSviewer 1.6.20 to create a keyword co-occurrence map (Figure 6), one can effectively visualize the research hotspot and direction of a particular field. To ensure accuracy, the minimum co-occurrence of keywords was set to 10, resulting in a total of 152 keyword co-occurrence maps. The keyword co-occurrence maps are categorized into five major groups. ① The green area represents research on neuropathic pain, which is often associated with oxidative stress, neuroinflammation, pain hypersensitivity, anxiety, depression, and cognitive-behavioral disorders. The treatment for nerve damage includes acupuncture, nerve catheters, and analgesic drugs such as nitric oxide, morphine, and cannabis. This treatment is mediated by cytokines, TNF-α, and NF-κB signaling. ② The red area represents research on nerve injury repair and regeneration, including Schwann cells, axonal regeneration, Wallerian degeneration, angiogenesis, and other mechanisms. It also covers therapeutic modalities such as 3D printing technology, drugs, electrical stimulation, nerve catheters, scaffolds, stem cells, and rehabilitation engineering. ③ The yellow area represents the animal experimental modeling approach, which involves establishing a rat model of sciatic nerve entrapment through surgical and pharmacological nerve block, as well as using total hip replacement surgery. ④ The blue area represents the treatment of muscle atrophy caused by sciatic nerve injury. Various methods are employed, including electrical stimulation, electroacupuncture, electrophysiology, exercise, magnetic resonance, mesenchymal stem cells, and exosomes. ⑤ The purple area represents the pathway of repairing sciatic nerve injury, which is studied from the perspective of proteomics, anti-inflammation, antioxidant, regulation of apoptosis, and autophagy. The map clearly shows that research on nerve regeneration and neuropathic pain forms a significant segment of the field. It is important to note that these five research directions are interrelated and not independent.

#### Keywords cluster

**Figure 7 fig7:**
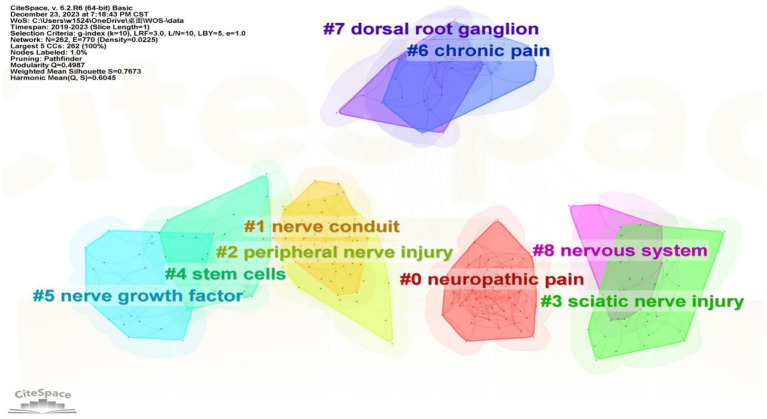
This diagram shows common citation clusters for research on sciatic nerve injury treatment from 2019 to 2023. Each color block represents a cluster group, with the cluster number being inversely proportional to the cluster size. For example, #0 represents the largest cluster.

The CiteSpace 6.2.R6 visualization software was used to perform keyword clustering network analysis. A keyword clustering map was generated using the log-likelihood ratio algorithm (LLS) after eliminating relevant subject words. 9 keyword clustering groups were obtained, as shown in Figure 7. It is generally accepted that a value of S > 0.7 indicates a convincing clustering, while Q > 0.3 indicates a significant cluster structure. The mapping shows a Mean Silhouette of 0.7673 and a Modularity Q of 0.4987, indicating good homogeneity among the keyword clusters and a satisfactory mapping effect. Table 6 displays the specific Silhouette value and Label (LLR). The research hotspot in this field in recent years is Neuropathic Pain, Nerve Catheterization, Peripheral Nerve Injury, and Stem Cells, which are the large research areas of the Keyword clustering. The English clustering labels can be classified into two categories. Category 1 pertains to sciatic nerve repair research (#2, #3, #5, #7, #8), which includes keywords such as nerve regeneration, schwann cells, functional recovery, nerve growth factor, neurotrophic factor, nerve conduit, pulsed radiofrequency, angioplasty, and dorsal root ganglion. Category 2 centers around sciatic analgesic treatment, including oxidative stress, nerve conduit, hyaluronic acid, chitosan nerve conduits, cannabidiol, tissue engineering, gene therapy, Schwann cell, and management. It has been found that chronic pain often causes emotional problems such as depression and anxiety and is associated with gender differences (11). Additionally, current research is increasingly focused on functional indices of nerve damage, tactile abnormalities related to pain, and other relevant indicators.

**Table 6 tab6:** Main clusters of keywords.

ClusterID	Size	Silhouette	Label (LLR, log-likelihood ratio)
#0	55	0.848	neuropathic pain (224.32, 1.0E-4); oxidative stress (83.93, 1.0E-4); nerve regeneration (59.27, 1.0E-4); chronic constriction injury (53.22, 1.0E-4);
#1	37	0.668	neuropathic pain (64.13, 1.0E-4); nerve conduit (40.69, 1.0E-4); nerve guidance conduit (31, 1.0E-4); hyaluronic acid (25.37, 1.0E-4); chitosan (21.96, 1.0E-4)
#2	36	0.853	peripheral nerve injury (93.49, 1.0E-4); schwann cells (44.63, 1.0E-4); nerve regeneration (40.25, 1.0E-4); functional recovery (39.62, 1.0E-4)
#3	35	0.736	sciatic nerve injury (52.22, 1.0E-4); system (20.08, 1.0E-4); sex differences (16.06, 1.0E-4); diagnosis (16.06, 1.0E-4); nerve guidance conduit (15.75, 1.0E-4)
#4	28	0.786	stem cells (46.22, 1.0E-4); neuropathic pain (41.08, 1.0E-4); mesenchymal stem cells (37.82, 1.0E-4); tissue engineering (27.2, 1.0E-4); transplantation (21.59, 1.0E-4)
#5	22	0.663	nerve growth factor (19.16, 1.0E-4); vascular endothelial growth factor (15.14, 1.0E-4); magnetic resonance imaging (11.08, 0.001); gene therapy (11.08, 0.001); angiogenesis (9.46, 0.005)
#6	22	0.701	chronic pain (38.2, 1.0E-4); management (32.84, 1.0E-4); cannabidiol (21.88, 1.0E-4); surgery (19.25, 1.0E-4); schwann cells (12.91, 0.001)
#7	18	0.733	dorsal root ganglion (36.71, 1.0E-4); neurotrophic factors (22.46, 1.0E-4); growth factor (18.2, 1.0E-4); pulsed radiofrequency (15.59, 1.0E-4); sensory neurons (14.25, 0.001)
#8	9	0.887	nervous system (22.14, 1.0E-4); spinal cord injury (19.24, 1.0E-4); angioplasty (14.26, 0.001); regional anesthesia (14.26, 0.001); schwann cells (10.36, 0.005)

#### Keywords burst

**Figure 8 fig8:**
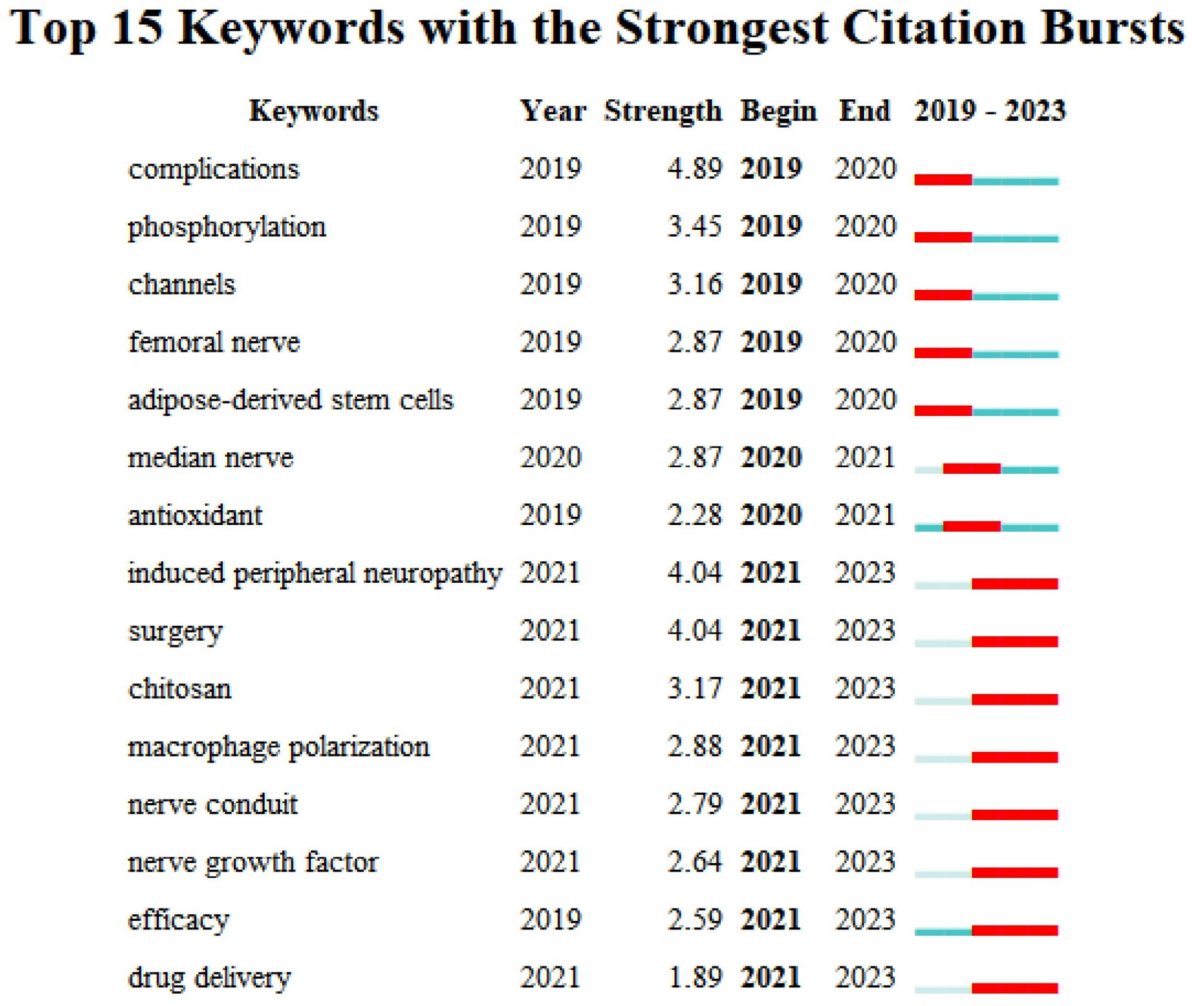
The diagram shows the top 15 keywords with the strongest citation bursts. The blue lines indicate the time intervals, and the red lines indicate the duration of the citation bursts.

CiteSpace6.2.R6 software was utilized to analyze the selected literature for burst words. Burst words are words with a high-frequency change rate in the corresponding time and can reflect the development trend of a certain research field. Figure 8 displays the top 15 keywords with the strongest citation bursts in published articles on treatments related to sciatic nerve injury, the keyword bursts are divided into two distinct phases. The main burst words during the first phase (2019–2021) included complications, phosphorylation, pathway, femoral nerve, adipose-derived stem cells, and antioxidants. This phase aims to repair damaged nerves through the use of antioxidants, stem cell transplantation, and regulation of the phosphorylation level of related protein kinases and signaling pathways to promote nerve regeneration. The main keywords for Phase 2 (2021–2023) include peripheral neuropathy, surgery, chitosan, macrophage polarization, nerve conduit, nerve growth factor, and drug delivery. Research in this field is currently focused on using drugs and chitosan nerve conduits to induce macrophage polarization, promote neovascularization, and synaptic plasticity to repair sciatic nerve defects (9, 13, 14).

### Journal

#### Cited journals

Based on the citation frequency of journals, one can determine the influence of a particular journal in the field of sciatic nerve injury repair. Data analysis reveals that between 2019 and 2023, publications related to this field were primarily featured in 118 different journals, with 11 of these journals receiving over 700 citations each, as detailed in Table 7. The *EXP NEUROL* has the highest citation frequency with 1,253 citations and the third-highest centrality ranking (0.24), following *BIOMATERIALS* (0.28) and *J NEUROSCI* (0.26). This journal has a significant impact in the field. The 11 top journals had an average impact factor of 6.02. *BIOMATERIALS* had the highest impact factor (IF = 14.0). All of the journals had more than 120 citations per year in sciatic nerve injury repair research, with a focus on bioengineering (15–18) and regenerative medicine (8, 10, 19).

**Table 7 tab7:** The top 11 cited journal.

Cited journals	Count	Centrality	2024-IF
EXP NEUROL	1,253	0.24	5.3
J NEUROSCI	1,201	0.26	5.3
PLOS ONE	1,164	0.05	3.7
PAIN	852	0.13	7.4
P NATL ACAD SCI USA	849	0.06	11.1
SCI REP-UK	849	0.02	4.6
NEURAL REGEN RES	833	0.07	6.1
BRAIN RES	804	0.1	2.9
NEUROSCIENCE	781	0.03	3.3
NEUROSCI LETT	758	0.02	2.5
BIOMATERIALS	729	0.28	14.0

#### Dual-map overlays showing the research directions of burst journals

**Figure 9 fig9:**
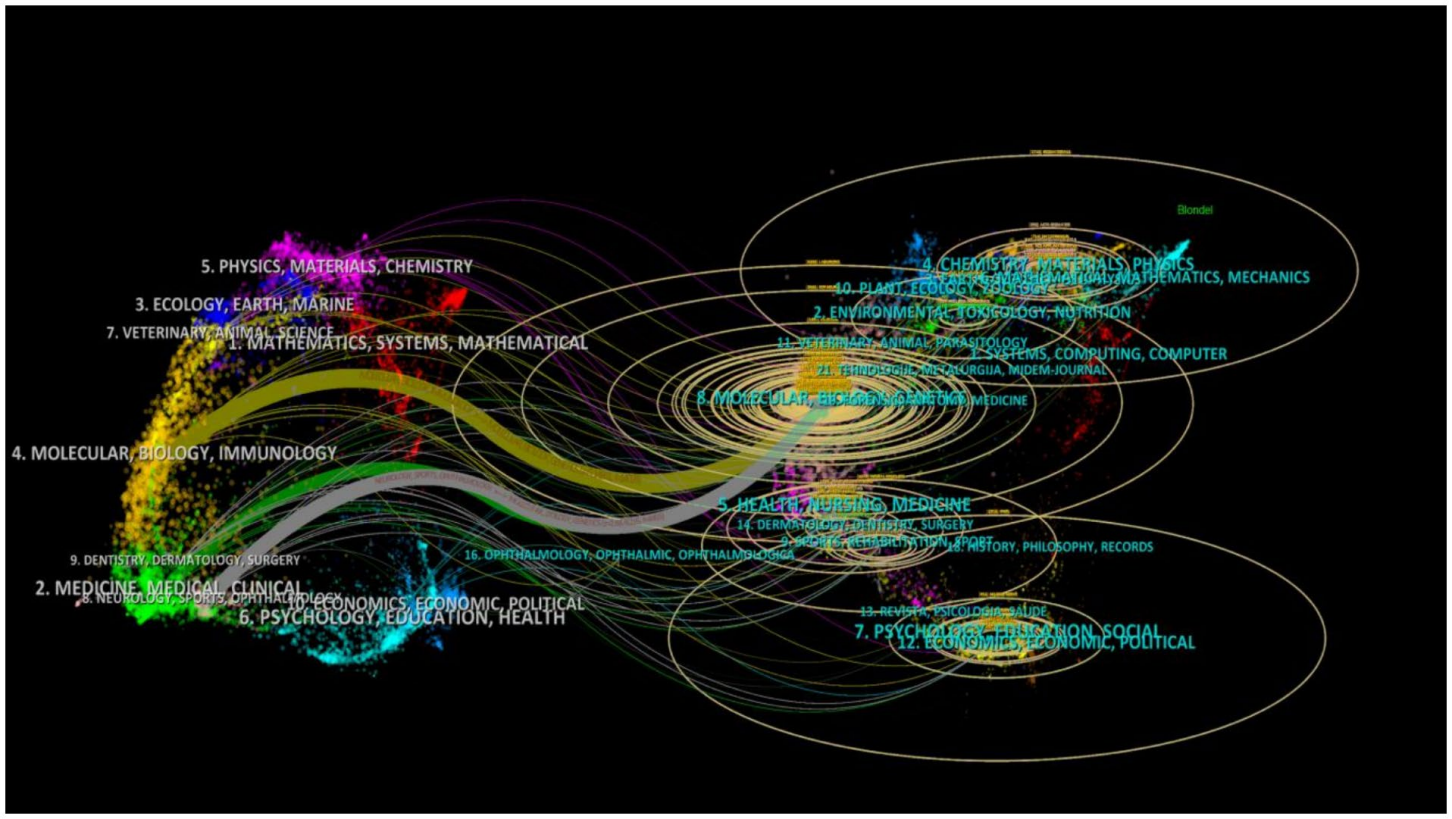
Dual-map overlays of cited/citing journals. Each point on the map represents a journal that has published research on therapeutic aspects related to sciatic nerve injury. The map is divided into two halves: the administering citation map on the left and the cited map on the right. The citation connectors provide interdisciplinary relationships between the fields. The z-Scores function highlights the smoother trajectories, with higher scores indicating thicker links. The ellipses represent the number of authors (length) and publications (width).

The journal map overlay illustrates the position of sciatic nerve injury research within the broader academic field. Figure 9 shows that a total of 231 journals have published such research. The figure shows that the fields of Molecular Biology, Immunology (yellow trajectory *z* = 9.14661, *f* = 14,098), Medicine, Clinical Medicine (green trajectory *z* = 1.9450856, *f* = 3,342), Neurology, Kinesiology, and Ophthalmology (gray trajectory *z* = 2.6876216, *f* = 4,455) are influenced by Molecular Biology and Genetics. The authors primarily studied sciatic nerve repair from the perspective of Molecular Biology and Immunology.

## References

### Co-citation analysis of references

Literature citation frequency is commonly used to evaluate the influence of a researcher or publication in a particular field. Table 8 summarizes the top 10 cited literature based on citation analysis. The study with the highest citation frequency (76) and an impact factor of 81.5 is “Neuropathi

**Table 8 tab8:** The ten references with the highest citation frequency indexed.

Title	Frist author	Counts	Centrality	IF	Journal
Neuropathic pain	Colloca L	76	0.12	81.5	Nature Reviews Disease Primers
The repair Schwann cell and its function in regenerating nerves	Jessen KR	67	0.14	5.5	Journal of Physiology-london
Nerve guide conduits for peripheral nerve injury repair: A review on design, materials and fabrication methods	Vijayavenkataraman S	67	0.07	9.7	Acta Biomaterialia
Mechanisms of Schwann cell plasticity involved in peripheral nerve repair after injury	Nocera G	59	0.02	8.1	Cellular And Molecular Life Sciences
The Success and Failure of the Schwann Cell Response to Nerve Injury	Jessen KR,	58	0.1	5.3	Front Cell Neurosci
Macrophage biology in the peripheral nervous system after injury	Zigrnond RE	58	0.26	6.7	Progress in Neurobiology
Peripheral nerve regeneration and intraneural revascularization	Caillaud M	51	0.01	6.1	Neural Regen ResNeural Regeneration Research
Current status of therapeutic approaches against peripheral nerve injuries: a detailed story from injury to recovery	Hussain G	44	0.21	9.1	International Journal of Biological Sciences
The wound microenvironment reprograms schwann cells to invasive mesenchymal-like cells to drive peripheral nerve regeneration	Clements MP	43	0.11	16.2	Neuron
Macrophage-induced blood vessels guide schwann cell-mediated regeneration of peripheral nerves	Cattin AL	43	0.01	64.5	Cell

c Pain Literature” by Colloca L, published in *Nature Reviews Disease Primers*. The study reviews the neurologic mechanisms behind chronic neuropathic pain and potential treatments (20). Furthermore, Jessen KR’s article, having been published twice and cited a total of 125 times, demonstrates significant influence in this research field. Additionally, five out of the top 10 highly cited articles focus on Schwann cells, highlighting their crucial role in sciatic nerve repair.

### Citation bursts analysis of reference

**Figure 10 fig10:**
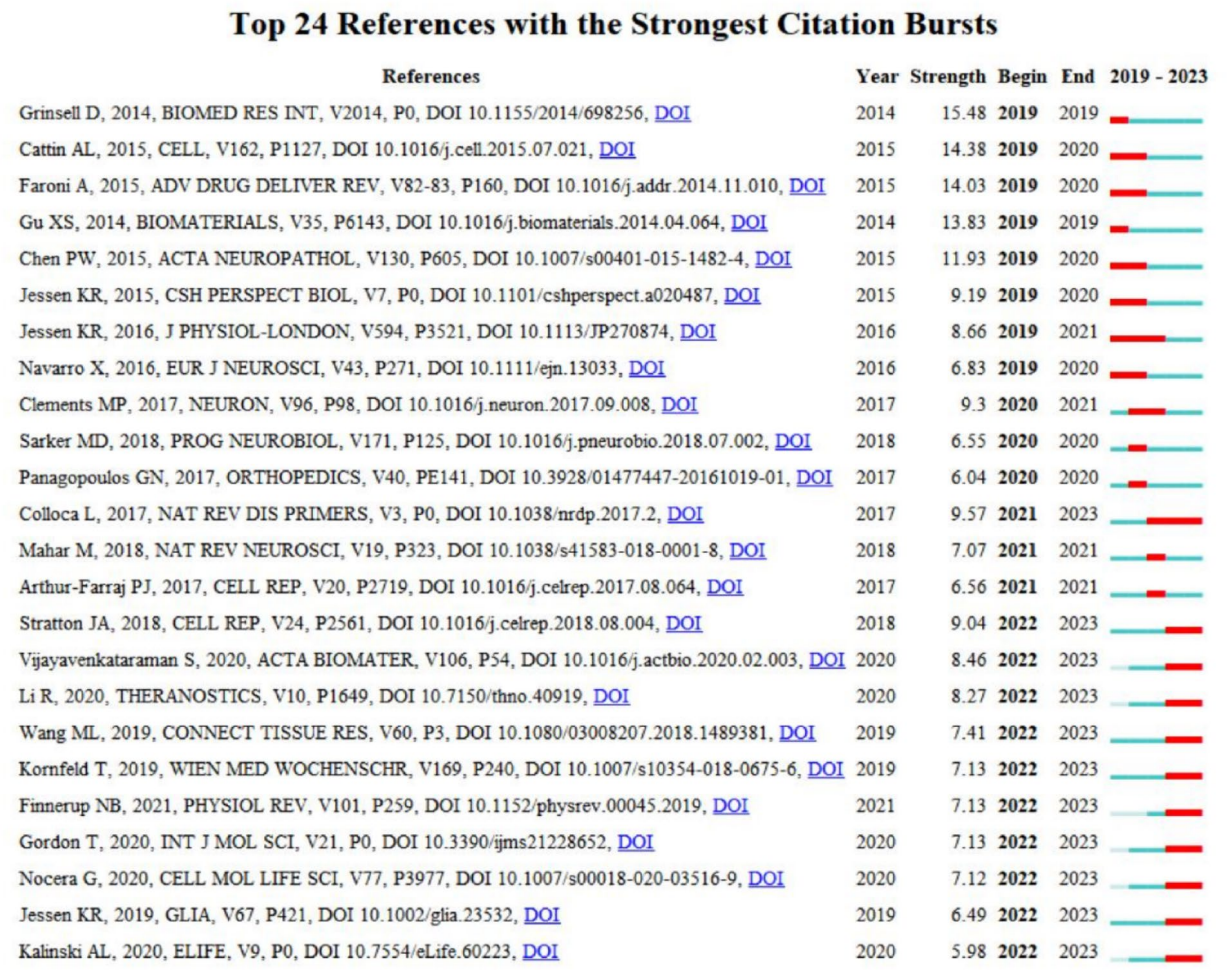
TOP 24 References with the strongest citation bursts.

The CiteSpace 6.2.R6 software was used to analyze reference highlighting, with the node set to Reference. The top 24 references in terms of citations were identified. The document titled “Peripheral Nerve Reconstruction after Injury: a Review of Clinical and Experimental Therapies” by Grinsell et al. (21) published in *Biomed Res Int*, had the highest highlighting strength of 15. According to reference (8), surgery or nerve autograft can significantly improve the outcome of proximal nerve injury. Additionally, fascicular-level nerve reconstruction is a better option. The article emphasizes the importance of enhanced surgical care to promote axonal regeneration, improve distal motion through electrical stimulation, and delay or avoid Waller’s degeneration to effectively resolve neurological deficits. Furthermore, Figure 10 illustrates that the majority of reference outbreaks have concluded by 2021 in terms of outbreak chronology. To date, 11 publications have been produced, focusing on the role and molecular mechanisms of Schwann cells, macrophages, and neurotrophic factors in reconstructing peripheral nerve fibers, promoting axon regeneration, and neurological recovery (22–26). Additionally, studies have focused on the mechanisms of neuropathic pain and potential therapies (20, 27, 28) discovered that cytosolic action plays a crucial role in nerve clearance, promoting an anti-inflammatory environment and regulating lesion-induced nerve repair. The literature, in which the subject term Schwann cell appeared several times, suggests that their involvement in regeneration and analgesia after sciatic nerve injury is a current research topic in this field.

## Discussion

In this paper, we use CiteSpace and VOSviewer software to visualize the Web of Science database and present visual maps of the number of articles, countries, institutions, authors, journals, references, and keywords. We then analyze these maps to extract data. The

study’s findings indicate that scholars domestically and internationally are highly interested in the repair and regeneration of sciatic nerves following injury. Over the past 5 years, 2,653 articles have been published on this topic. Furthermore, the number of articles published has increased steadily from 2019 to 2021, peaking in 2021. Although the number of articles published in 2021–2023 has decreased slightly, the overall number of articles issued in the year is more than 490. Additionally, the frequency of citations has increased substantially year by year. This suggests that research in this field will continue to be emphasized in the future. Moreover, a great majority of publications Document Typeswere articles, and only a few were reviews articles, according to the bibliometrics, suggesting that there is a continuing need for novel investigation at this stage. The most cited article is Repair-Schwann cell update: Adaptive reprogramming, EMT, and stemness in regenerating nerves, with 196 citations in the last 5 years and an impact factor of 6.2, which is significant in the field. The quality of the article is good. China and the United States are the countries with the highest number of publications, having published 939 and 552 documents, respectively, which accounts for 56.20% of the total number of publications in the past 5 years. The United States has a greater international impact, with a maximum centrality of 0.3, followed by China. Despite China having the highest number of publications, the overall quality is not sufficiently high. Therefore, Chinese researchers in this field should be encouraged to create high-quality articles to increase their international influence continuously.

Furthermore, an analysis of country and author co-occurrence revealed the formation of 16 collaborative teams. These teams were primarily composed of researchers from the same institution, affiliated hospitals, or region. Cross-province and cross-country studies are infrequent due to the lack of close connections between countries. Therefore, cooperation between countries in this field should be increased to establish a foundation for the treatment of clinical peripheral nerve injuries. Wang, Yu has the highest number of publications. His primary research is focused on neural tissue engineering. He uses self-assembled peptide nanofibers hydrogel combined with neurotrophic factors, as well as decellularized nerve catheters, nerve grafts, and Schwann cell grafts to promote peripheral nerve reconstruction and neovascularization (29–31). Yi Sheng’s team investigated the mechanism of Schwann cells, proteins, growth factors, and cytokines in repairing sciatic nerve injuries (32–35). Hussain and Ghulam’s third-ranked team published an article on modeling sciatic nerve injury through animal experiments in mice. The study found that certain plants and their derived compounds, such as the methanolic extract of *Foeniculum vulgare*, *Strychnos nux-vomica*, *Cannabis sativa* L., and *Moringa oleifera* Lam, possess anti-inflammatory, antioxidant, neuroprotective, and analgesic properties that promote the repair of peripheral nerves (4, 36, 37).

The study conducted by the issuing institutions revealed that the research advantage lies primarily with universities. Among the top 10 institutions, 7 are based in China, with Nantong University having the highest number of articles and a significant influence in the research field. Additionally, the study found that Chinese medicine universities have increasingly focused on the treatment of sciatic nerve injuries in recent years, indicating a growing interest in this field. Based on visual analysis of journals, *EXP NEUROL* has the largest number of document collections with a centrality of 0.21, indicating high influence and making it the mainstream journal in this field. Research in this field primarily focuses on Molecular Science, Biology, Immunology, Medicine, Clinical Medicine, Neurology, Kinesiology, and Ophthalmology.

Keyword co-occurrence and cluster can effectively summarize the main content of an article. An analysis of the keywords from the last 5 years reveals that neuropathic pain (414), expression (396), and Schwann cells (357) are the most frequently occurring hot keywords in this field. Neuropathic pain is a current research focus and a challenging field that significantly impacts patient’s physical recovery and can lead to adverse emotions such as anxiety and depression (38). Various treatments have been used to alleviate this type of pain, including pharmacological interventions (39–42), electrical stimulation (43, 44), exercise (12), nerve catheterization (45), and electro-acupuncture (46, 47).

The cluster map primarily revolves around the research progress in nerve regeneration following sciatic nerve injury alleviation of neuropathic pain muscle atrophy repair pathways and therapeutic mechanisms. Among these the most researched are Schwann cells which not only secrete signaling molecules to promote neuron survival and axonal regeneration but also activate local mesenchymal stem cells to migrate to the damaged tissue areas (48, 49).

The 15 keyword bursts were divided into two stages according to their mapping. In the first stage,(2019–2021), Complications had the highest burst strength of 4.89.Research indicates that diabetes mellitus is often complicated by sciatic nerve dysfunction and neuropathic pain (50–52) established a model of type I diabetes mellitus in rats by injecting streptozotocin into the intraperitoneal cavity. They found that exercise and insulin-like growth factor 1 treatment reduced the expression of vascular endothelial growth factor-A, platelet reactive protein-1, and nuclear factor-κB in the diabetic sciatic nerve, which promoted sciatic angiogenesis. In the second stage: From 2021 to the present, surgical treatments have exhibited the highest burst intensity, with a score of 4.04. These treatments mainly include nerve grafts (53, 54), fibrin glue (55–57), and nerve conduits (7, 58, 59, 60), indicating that surgical treatment is currently a popular research topic in the field of sciatic nerve repair and is predicted to continue to receive attention in the future.

## Conclusion

This study conducted a comprehensive review of the research in the field of SNI over the past 5 years. The analysis of the research hotspots and keywords revealed that the repair of sciatic nerve injury and analgesia are the current research hotspots and challenges. Additionally, the study found that nerve transplantation, nerve catheterization, and cellular therapies are the current and future possible research focuses or significant breakthroughs in this field. Notably, stem cell therapy has made significant progress in the repair of SNI. Stem cells have the capacity to differentiate into neuronal and glial cells, which can facilitate nerve regeneration following sciatic nerve injury. This approach has the potential to revolutionize the treatment of sciatic nerve injury, offering new avenues for future therapeutic strategies. Secondly, the role of trophic factors in the repair of sciatic nerve injury has garnered significant interest. These factors can promote distal nerve regeneration after nerve injury, offering novel insights and avenues for future research and clinical practice. In the treatment of neuropathic pain, non-surgical aspects of the last 2 years have focused on the exploration of electrical stimulation, electroacupuncture, and medication. It is predicted that these will continue to be investigated in the future as well, with the aim of developing new analgesic treatments. Among them, electrical stimulation therapy has been shown to promote the increase of neurofactors for axonal growth and to improve the speed and effect of nerve repair. This provides a new tool for clinical practice. In conclusion, the results of the advances in sciatic nerve injury research provide useful information for future research and clinical practice. In the future, it is necessary to continue to explore new treatments and technologies in depth in order to provide better treatment options for patients with sciatic nerve injury. At the same time, it is important that policymakers pay attention to the development of this field and provide the necessary support and safeguards for related research and treatment.

However, autologous nerve grafting, despite being non-immunogenic effects and containing nerve regeneration promoting factors such as Schwann cells, adhesion molecules and neurotrophic factors, has limitations such as limited tissue availability, loss of neurological function, scarring, and formation of neuromas. On the other hand, allogeneic nerve grafts are easily available and have excellent recovery, but they are costly and have many side effects. Nerve conduits can serve as an alternative therapy to autologous nerve grafts and provide an ideal microenvironment for neuronal recovery. Biodegradable materials, such as collagen, polylactic acid, chitosan, and hydrogel, are currently the most commonly used. However, the biocompatibility of these materials is sometimes reduced due to their xenobiotic properties. In contrast, cell therapy and plant-derived compounds accelerate nerve regeneration, with Schwann cells being the most widely used. Future research should explore the use of more desirable and reliable materials for nerve conduits, as well as the discovery or design of potent compounds capable of restoring injured nerves. These treatments could be combined with rehabilitation or drug therapies to seek optimal results.

### Limitations of the study

This study only selected literature from the Web of Science Core Collection Indexed Journal Database, which may introduce bias. To broaden the scope of the search and conduct more in-depth exploration in the future, strengthening talent exchange and regional cooperation is recommended for establishing a diversified research network. Additionally, this study’s limitations in language, literature type, and time period may have led to the exclusion of relevant literature, resulting in potentially biased data. Therefore, further improvements in methodology are necessar.

## Author contributions

YW (1st author): Writing – original draft, Writing – review & editing, Conceptualization, Data curation, Formal analysis, Investigation, Methodology, Project administration, Software. YahW: Writing – original draft, Data curation, Supervision. LL: Supervision, Writing – original draft, Data curation. TL: Supervision, Writing – original draft, Data curation. YW (5th author): Funding acquisition, Writing – review & editing, Project administration, Validation. FP: Investigation, Writing – review & editing, Formal analysis, Validation.
